# Non-Auxetic Mechanical Metamaterials

**DOI:** 10.3390/ma12040635

**Published:** 2019-02-20

**Authors:** Christa P. de Jonge, Helena M. A. Kolken, Amir A. Zadpoor

**Affiliations:** Faculty of Mechanical, Maritime and Materials Engineering, Delft University of Technology (TU Delft), Mekelweg 2, 2628 CD Delft, The Netherlands; h.m.a.kolken@tudelft.nl (H.M.A.K.); a.a.zadpoor@tudelft.nl (A.A.Z.)

**Keywords:** non-auxetic, mechanical metamaterials, lattice structures, volume-preserving materials, fatigue

## Abstract

The concept of “mechanical metamaterials” has become increasingly popular, since their macro-scale characteristics can be designed to exhibit unusual combinations of mechanical properties on the micro-scale. The advances in additive manufacturing (AM, three-dimensional printing) techniques have boosted the fabrication of these mechanical metamaterials by facilitating a precise control over their micro-architecture. Although mechanical metamaterials with negative Poisson’s ratios (i.e., auxetic metamaterials) have received much attention before and have been reviewed multiple times, no comparable review exists for architected materials with positive Poisson’s ratios. Therefore, this review will focus on the topology-property relationships of non-auxetic mechanical metamaterials in general and five topological designs in particular. These include the designs based on the diamond, cube, truncated cube, rhombic dodecahedron, and the truncated cuboctahedron unit cells. We reviewed the mechanical properties and fatigue behavior of these architected materials, while considering the effects of other factors such as those of the AM process. In addition, we systematically analyzed the experimental, computational, and analytical data and solutions available in the literature for the titanium alloy Ti-6Al-4V. Compression dominated lattices, such as the (truncated) cube, showed the highest mechanical properties. All of the proposed unit cells showed a normalized fatigue strength below that of solid titanium (i.e., 40% of the yield stress), in the range of 12–36% of their yield stress. The unit cells discussed in this review could potentially be applied in bone-mimicking porous structures.

## 1. Introduction

During the last few years, the concept of “mechanical metamaterials” has gained much attention [1,2]. Mechanical metamaterials are materials for which the macro-scale properties are determined by the small-scale topological design [1,2]. Rationally designing the micro-architecture of these materials results in unusual or unique combinations of mechanical properties that are rarely seen in nature. Some of these exceptional mechanical properties include a negative Poisson’s ratio or a combination of low stiffness and high strength [3,4]. Recent developments seen in the field of additive manufacturing (AM, three-dimensional (3D) printing), have boosted the fabrication of mechanical metamaterials by facilitating a precise control over their micro-architecture. Therefore, a good understanding of the relationship between the topological design of the repeating unit cell and their macro-scale properties is highly relevant.

Mechanical metamaterials can be divided into two main categories depending on whether their Poisson’s ratio is positive or negative. Mechanical metamaterials with a negative Poisson’s ratio are better known as auxetic metamaterials, and have received a lot of attention during the last few years [5,6,7] including a few dedicated review articles [3,8,9]. Mechanical metamaterials with a positive Poisson’s ratio (i.e., conventional or non-auxetic metamaterials) have not received as much attention, and no comprehensive review article exists regarding their topology-property relationships. However, recent research has shown the importance of lattice structures with positive Poisson’s ratios in several areas including the design of lightweight structures [10,11], development of biomedically-relevant metamaterials (or meta-biomaterials [12,13]), and rational design of actuators for soft robotic applications [14]. In many of these application areas, lattice structures with positive Poisson’s ratios are not only important in their own right but also in combination with auxetic metamaterials [15,16,17]. Therefore, the aim of this paper was to review the topology-property relationships of non-auxetic mechanical metamaterials in general and five designs of such materials in particular. These designs include the lattice structures based on the diamond, cube, truncated cube, rhombic dodecahedron, and truncated cuboctahedron unit cells. The topology-property relationship has been extensively studied, using experimental [12,18], computational [19,20], and analytical [21,22] techniques. More specifically, we will focus on the lattice structures made from the high strength titanium alloy (Ti-6Al-4V), because many of the relevant studies use this particular alloy. Moreover, focusing on one single material means that the effects of topological design on the mechanical properties could be studied in isolation from those of the material type [23].

Given the fact that Ti-6Al-4V is an important biocompatible material often used in orthopedic implants, we payed special attention to the biomedical applications of these non-auxetic metamaterials [24,25]. Indeed, metamaterials made from titanium and its alloys (e.g., Ti-6Al-4V) have received increasing attention due to their potential application as bone substitutes [26,27]. Therefore, we could speak of a special class of metamaterials, otherwise known as meta-biomaterials [4,28]. These meta-biomaterials show an unusual combination of mechanical (e.g., stiffness), biological (e.g., tissue regeneration performance), and physical (e.g., diffusivity and permeability) properties [4]. Therefore, meta-biomaterials could mimic bone, which would reduce the mechanical mismatch at the bone-implant interface through a more uniform stress/strain distribution, reducing the risk of implant loosening [29]. The topologies that will be discussed in this review, could potentially be applied in such bone-mimicking meta-biomaterials. By having more knowledge about the topology-property relationship, one could simply adjust the macro-scale properties of a mechanical metamaterial by modifying its micro-architecture. Functionally grading the design of such architected materials may even enhance their advantages, since the architecture of bone is hierarchical and consists of a dense outer layer (cortical bone) and a more porous interior (trabecular bone) [30,31,32].

The following paragraphs will discuss the quasi-static mechanical properties and fatigue behavior of the mechanical metamaterials whose topological design is based on each of the above-mentioned unit cell types. We compared these mechanical properties according to the topology of the unit cells and their deformation mechanism(s). Other factors, such as the effects of the AM process, were also considered.

## 2. Topological Designs

### 2.1. Diamond

**Figure 1:**
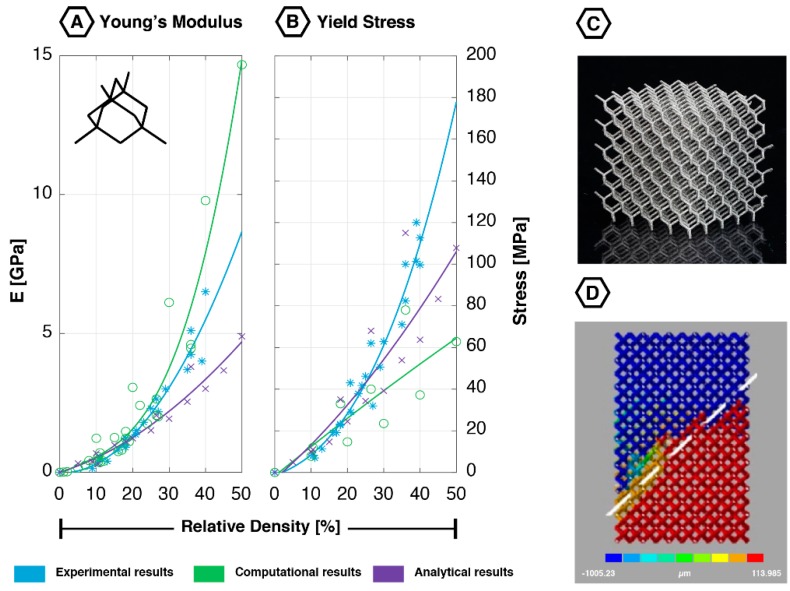
Mechanical properties of the diamond unit cell based on experimental, computational, and analytical data: (**A**) Young’s modulus [18,19,21,33,34,35,36,37,38,39,40,41,42]; (**B**) yield stress [18,19,21,33,34,36,37,38,40]; (**C**) additively manufactured diamond lattice (5 × 5 × 5 unit cells); (**D**) fatigue failure surface (45-degree shear band) showing the deformation in the loading direction; reprinted with permission from Ref. [20] © 2016 Elsevier.

The diamond unit cell (Figure 1) has 16 equal edges and 14 vertices [33]. Each node is attached to the four nearest nodes with an enclosed angle of 109.5° [33]. The diamond is one of the relatively new unit cells used in AM lattices with isotropic mechanical properties [33]. The struts in this unit cell mainly deform through bending [19].

#### 2.1.1. Quasi-Static Mechanical Properties

The mechanical properties of the diamond have been extensively studied in finite element (FE) and experimental studies. It has consistently shown a low Young’s modulus, yield stress and first maximal stress throughout the complete range of relative densities (Figure 1). The absolute Poisson’s ratio of the diamond geometry has only been studied using computational and analytical techniques. Values ranging from 0.45 to 0.58 have been reported [19,33,42]. An increment in the relative density is accompanied by a reduction in the absolute value of the Poisson’s ratio [19,33].

Several studies have investigated the effects of certain geometrical parameters (e.g., strut length, strut diameter, strut cross-section type, and cell size) on the mechanical properties of the diamond lattices [18,19,35,37,39]. FE simulations showed no significant effects of the strut length considering a constant relative density [19]. The cross-sectional shape of the strut, on the other hand, has been found to significantly affect the yield stress. A circular cross-sectional strut shape resulted in a significantly higher yield stress compared to the square and triangular cross-sectional shapes. In contrast, no effect has been observed on the Young’s modulus [19]. The effect of the strut diameter on the mechanical behavior of the diamond-based lattice has also been widely studied [18,19,35,39,42]. FE solutions showed that a reduction in the ratio between the cell size and the strut diameter leads to an increase in the Young’s modulus [39]. The absolute values of the dimensions did not seem to have an influence, since two samples with the same ratio and different size dimensions were found to have the same Young’s modulus [39]. Hedayati et al. (2017) found that increasing the relative density (by increasing the strut diameter) from 10% to 30% results in an increase in the Young’s modulus of 573%. The same increment in the relative density resulted in an increase in the yield stress of 407% [19].

When comparing the analytical, computational, and experimental data on the diamond lattices, one finds that the results obtained using these different approaches correspond very well for all mechanical properties (Young’s modulus, yield stress, first maximal stress), especially for low values of relative density (i.e., below 25%) (Figure 1).

#### 2.1.2. Fatigue Behavior

Numerous studies have investigated the fatigue behavior of AM diamond lattices [12,18,20,34,38]. Similar to many other porous structures, a three-stage strain accumulation pattern could be observed. This correlates well with the fatigue behavior found in earlier studies on cellular structures fabricated using Electron-beam Melting (EBM) and sintering techniques [38,43]. In the first stage, there is an initial increase in strain of which only a small part concerns plastic deformation. In stage II, this strain shows a plateau, where the first fatigue cracks are initiated. In stage III, a second increment in the strain can be recognized, in which the struts are plastically deformed, and the specimen finally fails. A layer-by-layer collapse is observed at the moment of failure, which eventually leads to a shear band of 45° around the specimen (Figure 1D) [20,34,35,38,40]. FE simulations have shown that at the onset of plastic deformation, struts were mainly deformed by bending [40]. Both tensile and compressive stresses have been found in the struts of the diamond lattices tested under compressive-compressive cyclic loading [34]. These stresses were equally distributed across the struts [42]; however, tensile stresses are probably the cause of the final failure [34]. A positive correlation was found between the strain accumulation locations and the location of the final fracture [35], whereas a change in the applied stress ratio (minimum compressive stress divided by the maximum compressive stress) did not change the failure appearance [34].

Most of the fatigue studies have investigated the effects of the relative density, applied stress, and applied stress ratio on the fatigue life of diamond lattices [12,34,38]. The relative density has shown to exhibit a positive correlation with the structure’s fatigue life, just like the applied stress ratio [20,34]. The absolute applied stress level, in contrast, showed a negative correlation [20,34,38]. The endurance limit has been found to range between 15 and 32% of the yield stress, depending on the porosity of the structure [12,34,38].

### 2.2. Cube

**Figure 2:**
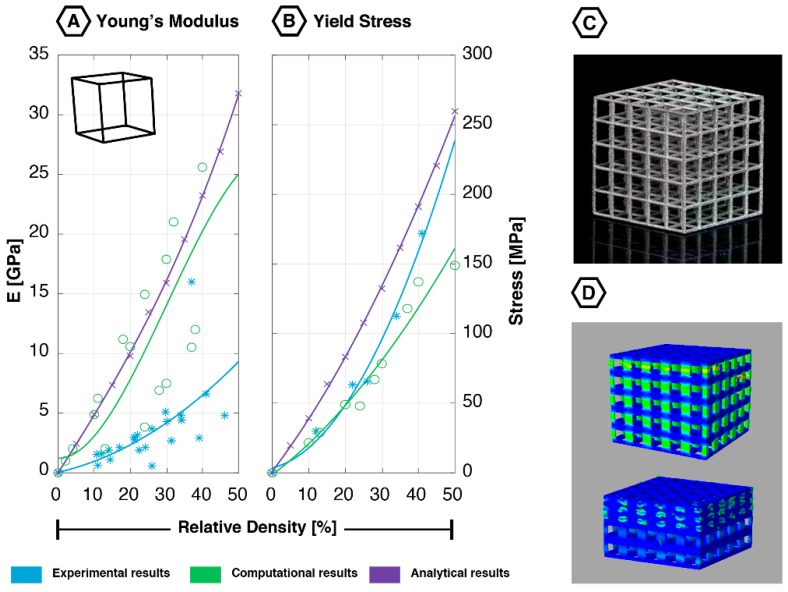
Mechanical properties of the cubic unit cell based on experimental, computational, and analytical data: (**A**) Young’s modulus [18,19,21,40,41,42,44,45,47,48,49,50]; (**B**) yield stress [18,21,40,47,48]; (**C**) additively manufactured cubic lattice (5 × 5 × 5 unit cells); (**D**) start and end stage of the deformation procedure showing a layer-by-layer collapse; reprinted with permission from Ref. [40] © 2015 Elsevier.

The cubic unit cell (Figure 2) is a straightforward geometry, with 12 equal edges and eight vertices. Each one of the vertices is connected to three edges, all enclosing a 90-degree angle with their neighbor. This unit cell is symmetrical in all directions, resulting in isotropic mechanical behavior. Due to this configuration, none of the struts parallel to the direction of loading will bend; instead, they deform through compression (or buckling). The struts aligned normal to the loading direction will translate [19,40,41,44,45]. When rotating the cubic unit cells by an angle of 45°, the struts mainly deform by bending [44]. The deformation mechanism therefore evolves from compression to bending when decreasing the angle of the applied load relative to the horizontal axis [44].

**Figure 3:**
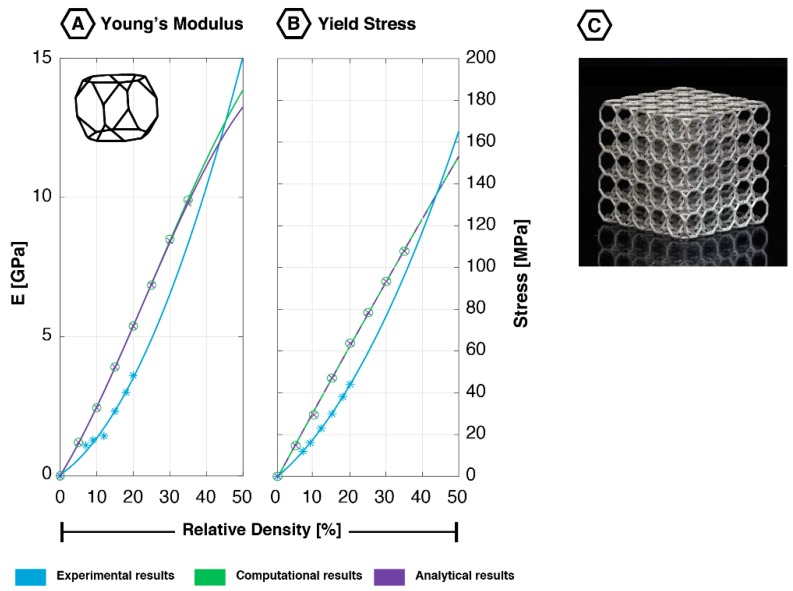
Mechanical properties of the truncated cube unit cell based on experimental, computational, and analytical studies: (**A**) Young’s modulus [18,19]; (**B**) yield stress [18,19]; (**C**) additively manufactured truncated cubic lattice (5 × 5 × 5 unit cells).

The topology of the truncated cube (Figure 3) is fairly similar to the cubic structure, and therefore, will also be discussed in this section. This unit cell, also known as the truncated hexahedron, is an Archimedean solid. It consists of 36 edges, 24 vertices, and 14 regular faces (eight triangular and six octagonal). It has inclined and uninclined (i.e., vertical or horizontal) struts. The truncated cube is an isotropic geometry and its struts mainly deform through compression [46].

#### 2.2.1. Quasi-static Mechanical Properties

Structures built from the cubic unit cell have shown to be very stiff, even at low values of relative density (Figure 2) [18]. Other properties, such as the yield stress and first maximal stress, are similarly high [12,18,19,42,45,51]. The cubic lattice also shows a remarkable increase in the plateau to yield stress ratio, as well as its plateau to first maximal stress ratio as the relative density increases [18]. The absolute Poisson’s ratio of the cubic lattice is small, ranging from 0.089 to 0.190 for a range of apparent densities. In some cases, it approaches zero [19,41,44]. This can be explained by the aforementioned deformation mechanism, which lacks bending [19,44]. The strut thickness has been the only geometrical parameter studied for its effect on the compressive properties of the cubic lattice. Parthasarathy et al. (2010) showed that the compressive strength of the cubic lattice is positively correlated with the strut thickness, regardless of the relative density [50]. The overall relative density itself is also positively correlated with the compressive properties [12,18,19,45,50]. A study performed by Hedayati et al. (2017) has shown that increasing the relative density (by increasing the strut thickness) from 10% to 30% results in an increment of 218% in both yield stress and Young’s modulus [19].

In theory, the cubic unit cell is isotropic; however, the AM process introduces a certain level of anisotropy to the structure. This can be explained by the fact that horizontally oriented struts show inferior quality as compared to the vertically oriented struts [36]. Two studies support this effect, in which a cubic lattice was tested at a 90-degree angle from the build direction. As a result, a significantly lower Young’s modulus and yield stress were found compared to the values found for the lattices tested in the build direction [48,49]. In general, cubic lattices exhibit large variations in their bending and compressive Young’s modulus (maximum of 83.3%) [42].

The data obtained using computational and experimental techniques corresponds very well for both the first maximal stress and yield stress (for small relative densities in Figure 2) [12,44,45,47,48,49,52]. However, large deviations were found in the analytically obtained values. Regarding the Young’s modulus, both computational and analytical data do not correspond well with the experimentally obtained values (Figure 2).

Although the cube and truncated cube are somewhat alike, their mechanical properties are certainly not. Their Young’s moduli vary significantly for larger values of relative density (Figure 2 and Figure 3), with the cube being about twice as stiff according to certain computational and analytical studies. However, in experiments, the truncated cube exhibited a higher Young’s modulus [18]. This can probably be explained by the increased number of junction points, which means the stresses will be more uniformly distributed across the structure [18]. This effect is even more pronounced at higher values of relative density, since the truncated regions become denser, thus, leading to an even better stress distribution [18].

#### 2.2.2. Fatigue Behavior

The deformation seen in the cubic lattice’s elastic region is primarily dominated by the uniform compression of vertically oriented struts, followed by a layer-by-layer collapse until complete failure (Figure 2D) [40]. At failure, the cubic lattice demonstrates a shear band perpendicular to the loading direction [40,44,45,51]. The literature is not consistent about the location of the first failure [40]. Kadkhodapour et al. (2015) found that the top layer was the first to collapse, followed by the other layers from top to bottom [40]. Choy et al. (2017) argued that the first collapse occurred at the edges of the geometry [48], while Cuadrado et al. (2017) indicated that the first collapse occurred at a random location in the structure [44].

In the study performed by Yavari et al. (2015), none of the cubic samples (regardless of their relative density) failed after 10^6^ loading cycles during compression-compression fatigue tests, not even at 80% of their yield stress [12]. Zhao et al. (2016) found an endurance limit of 0.48 times the plateau stress [51]. However, the anisotropic behavior introduced by the AM process does change the structure’s fatigue strength when applying the load at different angles [36].

### 2.3. Rhombic Dodecahedron

**Figure 4:**
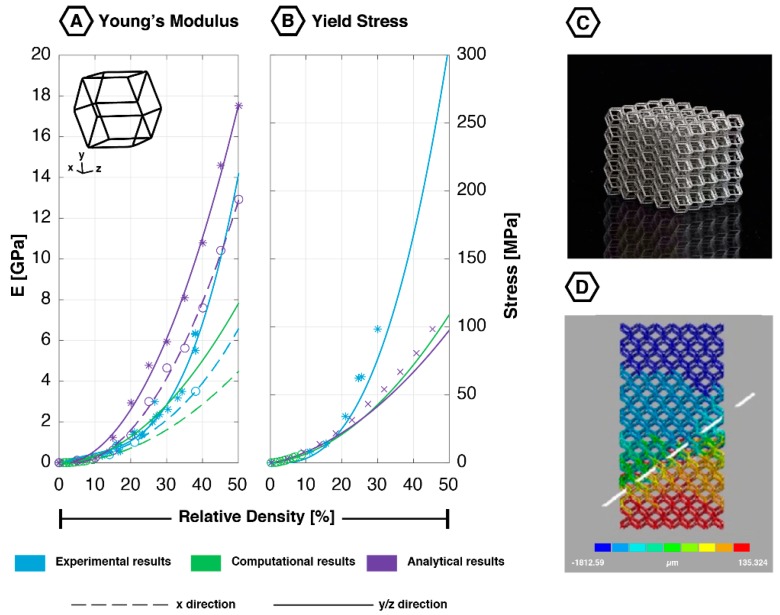
Mechanical properties of the rhombic dodecahedron unit cell based on experimental, computational, and analytical data: (**A**) Young’s modulus [12,18,19,21,22,45,51,55]; (**B**) yield stress [18,21,22]; (**C**) additively manufactured rhombic dodecahedron lattice (5 × 5 × 5 unit cells); (**D**) fatigue failure surface (45-degree shear band) showing the deformation in the loading direction; reprinted with permission from Ref. [20] © 2016 Elsevier.

The rhombic dodecahedron (Figure 4), sometimes referred to as rhomboidal dodecahedron, is a space-filling convex polyhedron [22]. This unit cell has 24 equal edges that are connected through 14 vertices, finally forming 12 identical parallelogram shaped faces [22]. The rhombic dodecahedron shows transversely isotropic behavior, which means its properties are different in one of its directions compared to the other two [22,53,54]. The struts in the rhombic dodecahedron mainly deform through bending [22,45].

#### 2.3.1. Quasi-Static Mechanical Properties

Due to its transversely isotropic behavior, the rhombic dodecahedron exhibits a significantly higher Young’s modulus in the y-/z-directions as compared to the x-direction (Figure 4) [19,22]. This difference increases significantly as the size of the unit cell decreases [53]. In contrast, the rhombic dodecahedron’s yield stress and first maximal stress appear to be isotropic (Figure 4) [19,22]. All of these compressive properties have been found to increase with relative density [12,18,19,54,55,56]. A study performed by Hedayati et al. (2017) showed that increasing the relative density (by increasing the strut thickness) from 10% to 30%, results in a 571% increment in the Young’s modulus. The same increase in the relative density resulted in an increase in the yield stress of 340% [19].

Conflicting results were found for the Poisson’s ratio. Babaee et al. (2012) found anisotropic values of 0, ±0.5 and ±1.0 for ν_yz_, ν_xz_ and ν_yx_, respectively [22]. On the other hand, Hedayati et al. (2017) built an FE model and tested all six orientations, which resulted in two deviating Poisson’s ratios, pointing at its transversely isotropic behavior [19]. Unlike the compressive properties, the Poisson’s ratio of this structure is not directly related to its relative density. Instead, Hedayati et al. (2017) presented a more concave relation [19].

Numerous studies have investigated the effects of certain geometrical parameters (strut length and strut cross-sectional shape) on the mechanical behavior of the rhombic dodecahedron lattice structure [19,57,58]. FE simulations showed no significant effect of the strut length for a constant value of the relative density [19]. Strut cross-sectional shape showed to have a significant effect on the yield stress, while it only slightly affected the Young’s modulus [19]. A circular cross-section resulted in a significantly higher yield stress as compared to the square and triangular shaped cross-sections [19].

The analytical and computational models have been successful in characterizing the mechanical behavior of the rhombic dodecahedron. At small values of relative density (<15%), the Young’s moduli and yield stresses have been found to overlap the experimentally derived values (Figure 4). However, at higher values, the results start to diverge. The computational results for the Young’s modulus underestimated the experimental values, whereas the analytical results consistently overestimated them (Figure 4). These differences have been found both in the x- and y-/z-directions. As compared to the analytical models, FE models could better predict the experimentally measured stiffness values once additive manufacturing irregularities were considered [41]. FE studies have shown that manufacturing irregularities reduce the mechanical properties of AM structures, especially for the lower values of relative density [19,41]. A reduction of 10–20% has been found for the Young’s modulus with variations caused by the different loading directions [41,54].

#### 2.3.2. Fatigue Behavior

During compression-compression fatigue testing, all rhombic dodecahedron samples failed before 10^6^ loading cycles, even for low stress values (20% of the yield stress) [59]. Experiments and FE models (with/without AM irregularities) showed the formation of a shear band at ±45° from the loading direction (Figure 4D) [20,51,54,55]. An FE model implementing the manufacturing irregularities predicted the failure pattern to accumulate around the initially failed struts [54]. Zhao et al. (2016) used Scanning Electron Microscopy (SEM) to observe the fracture surface after experimentally determining the fatigue life. Those observations showed fatigue striations on the fracture surface [51]. All observations support the fact that the rhombic dodecahedron has a fairly low fatigue strength, which was confirmed by an endurance limit of 0.12 times the yield stress after 10^6^ loading cycles [59]. This endurance limit was retrieved by extrapolating the power law, since all samples failed before reaching 10^6^ loading cycles [59]. These small values of the fatigue strength might be explained by the low radius to length ratio of the struts in this lattice. A lower ratio results in a smaller moment of inertia, leading to higher bending stresses, which in turn result in a lower fatigue strength [20]. The normalized fatigue S–N curves of rhombic dodecahedron geometries with different relative densities were similar and conform a power law (R² = 0.94) [59]. Similar to its compressive properties, the fatigue life of these structures increased with augmenting the relative density [20,56,59]. For stress levels below 60% of the yield stress, computational and experimental studies yielded similar results. Above this stress level, the computational prediction was significantly lower than the experimentally determined fatigue life. Once again, the irregularities of the AM process have been proven very important to be considered in an FE model. Failing to include this extra AM dimension, this may result in a 30–70% overestimation of the fatigue life of such structures [54].

### 2.4. Truncated Cubuctahedron

**Figure 5:**
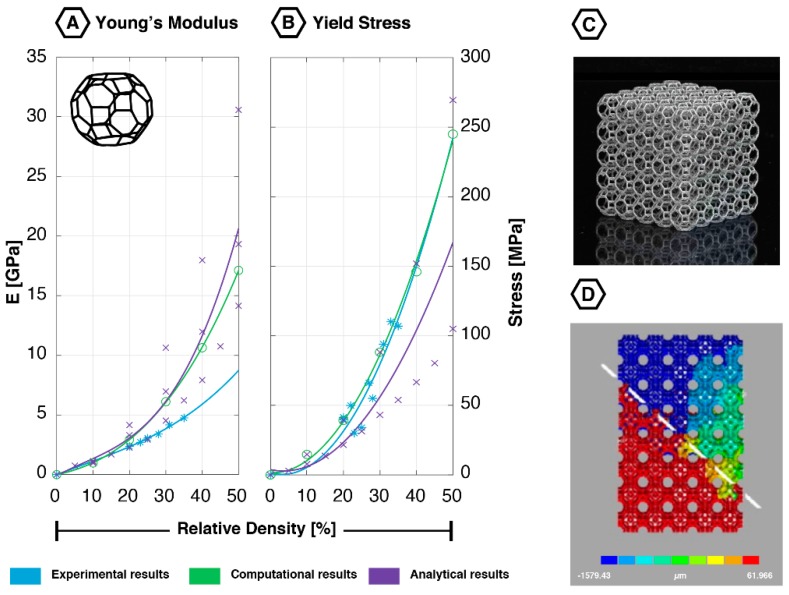
Mechanical properties of the truncated cuboctahedron unit cell based on experimental, computational, and analytical data: (**A**) Young’s modulus [18,19,21]; (**B**) yield stress [12,18,19,21,60]; (**C**) additively manufactured truncated cuboctahedron lattice (5 × 5 × 5 unit cells); (**D**) fatigue failure surface (45-degree shear band) showing the deformation in the loading direction; reprinted with permission from Ref. [20] © 2016 Elsevier.

The truncated cuboctahedron (Figure 5), also known as the rhombi cuboctahedron or rhombi truncated cuboctahedron, is an Archimedean solid structure. This unit cell has 72 edges that are connected through 48 vertices, forming 12 square-, eight regular hexagonal-, and six regular octagonal shaped faces [60]. In contrast to the other geometries in this review, literature on the mechanical properties of this unit cell is scarce.

#### 2.4.1. Quasi-Static Mechanical Properties

The truncated cuboctahedron falls in the category of structures with a relatively high stiffness at low values of relative density (<20%) [18], which makes this geometry particularly useful in lightweight structures. Additionally, it has been found that when the relative density increases, so does the ratio between the plateau and yield stress [18]. The truncated cuboctahedron seems to be a unit cell that is relatively easy to manufacture using AM techniques. Once printed, this lattice shows a limited number of imperfections and notches [12].

The data obtained on the truncated cuboctahedron using computational and experimental techniques correspond very well for the yield stress (Figure 5). Analytical results start to deviate from the latter for higher values of relative density (>15%). Regarding the Young’s modulus, both analytical and computational results correspond very well with experimental observations for the low values of relative density (<15%) (Figure 5). However, for larger values of relative density, both techniques overestimate the experimental results. Analytical and computational models found the Poisson’s ratio to vary between 0.13 and 0.49 [21,46].

#### 2.4.2. Fatigue Behavior

Like many other lattice structures, the truncated cuboctahedron shows a 45° shear band after failure (Figure 5D) [20]. Both experimental and numerical results showed that the truncated cuboctahedron exhibits a high fatigue strength, resulting in an endurance limit of 0.36 times the yield stress at 2 × 10^5^ loading cycles [12]. This is due to the relatively low bending stress in the struts of this geometry, caused by the high radius to length ratio [20]. The fatigue life of this lattice was found to be highly dependent on the relative density [12].

## 3. Discussion

The mechanical properties of Ti-6Al-4V additively manufactured (AM) porous structures have been broadly studied in the last decade using experimental, computational, and analytical methods. In the previous sections, we reviewed the quasi-static and fatigue properties of non-auxetic mechanical metamaterials built using diamond, cube, truncated cube, rhombic dodecahedron, and truncated cuboctahedron unit cells. In this section, we will discuss their topology-property relationship and their potential use in biomedical applications. Other applications will also be considered together with the recommendations for future research.

**Figure 6:**
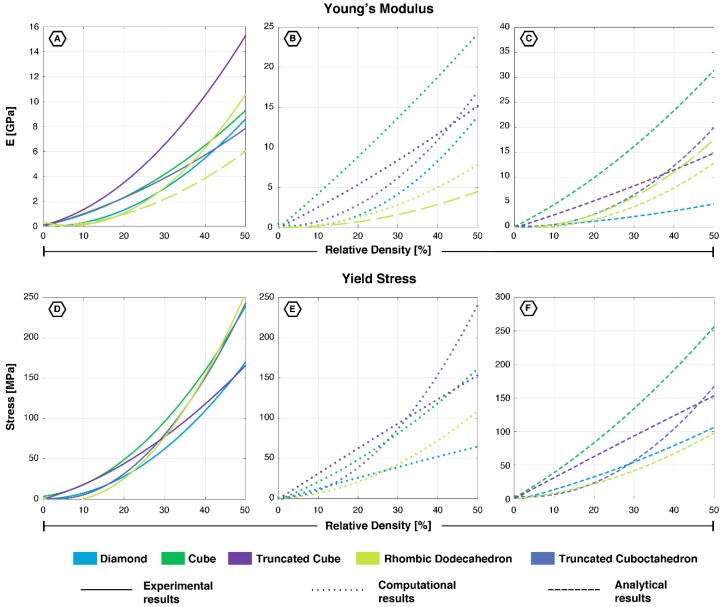
Young’s moduli (**A**–**C**) and Yield stresses (**D**–**F**) of all five unit cells based on experimental, computational, and analytical data. Since the rhombic dodecahedron is anisotropic, two lines have been displayed showing its properties in the x- (dashed line) and y-/z-directions. (**A**) [12,18,33,34,35,36,37,39,44,45,47,48,49,50,51,55]; (**B**) [19,33,39,40,41,42]; (**C**) [19,21,33]; (**D**) [12,18,33,34,36,37,38,40,47,48]; (**E**) [19,22,40]; (**F**) [19,21,33].

**Figure 7:**
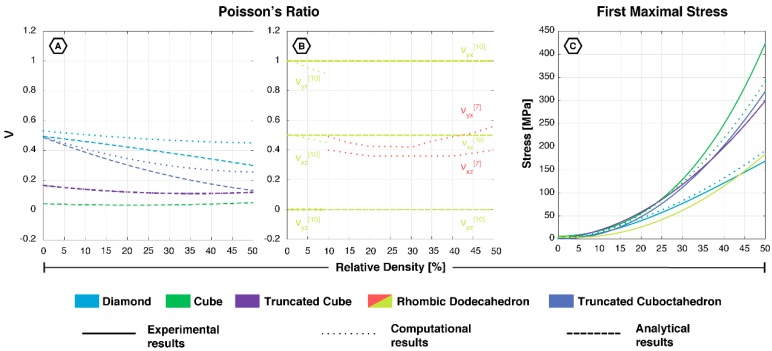
(**A**) Poisson’s ratio of the diamond, cube, truncated cube and truncated cuboctahedron and (**B**) rhombic dodecahedron; (**C**) first maximal stress of all five unit cells, based on experimental [12,18,33,34,35,36,37,39,44,45,47,48,49,50,51,55], computational [19,22,33,40,42], and analytical data [19,21,22,33].

Several studies have compared the mechanical behavior of these unit cells to one another, which have been presented in Figure 6 and Figure 7. Of all unit cells studied, the diamond consistently showed the lowest Young’s modulus, yield stress, and first maximal stress throughout the complete range of relative densities [18,19]. There were only two exceptions, concerning the experimentally derived values for the rhombic dodecahedron. This unit cell exhibited a lower Young’s modulus (both directions) and a lower first maximal stress for low values of relative density (<25%) [18]. The yield stress of the rhombic dodecahedron was somewhat lower than the truncated cuboctahedron, while their values started to overlap at higher values of relative density [18,19]. The cube, on the other hand, possessesed the highest yield stress and first maximal stress in all studies (Figure 6 and Figure 7) [12,18,19,42,45,51]. In terms of the Young’s modulus, the cube was surpassed by the truncated cube, which has been shown to be the stiffest unit cell studied [18]. The truncated cuboctahedron fell in the same category as the aforementioned two geometries for structures with a high stiffness at low values of the relative density (<20%) [18]. Its Young’s modulus was slightly smaller than the cube, while its yield stress ended up being comparable to the values found for the rhombic dodecahedron and the diamond at low values of the relative density (<20%).

Although limited data is available on the Poisson’s ratio of these non-auxetic mechanical metamaterials, some comparisons could be made. The rhombic dodecahedron has shown the most remarkable behavior, which due to its transversely isotropic character shows values (ν_yx_) reaching far beyond the isotropic limit of 0.5 (Figure 7) [19,22,61]. Therefore, it outperformed the other geometries, showing the biggest ratio of lateral contraction to axial stretch. However, in one of the other directions (ν_yz_) it showed the smallest Poisson effect, similar to that found for the cube [19,22,42]. In contrast, Hedayati et al. (2017) found a Poisson effect more closely resembling the reported values for the diamond [19,21,33,42]. Clearly, there is no consensus about the uniformity of the structure’s orientations. Therefore, experimental studies are essential to fully assess the deformation behavior of the rhombic dodecahedron, especially those considering all of the structure’s orientations.

Despite the considerable amount of data on the quasi-static mechanical properties of the lattice structures under discussion, there is only limited data available on their fatigue behavior. This can probably be explained by the high cost and time needed to perform a fatigue study. The available studies have shown that the fatigue behavior of porous biomaterials strongly depends on the relative density, applied stress, applied stress amplitude, material type, and the type of unit cell. Fatigue life decreased in the following order: cube, truncated cuboctahedron, diamond, and rhombic dodecahedron [12,13,20,34,38,51,54,56,59]. While data on the truncated cube is missing, we assume that this unit cell belongs in between the cube and the truncated cuboctahedron in terms of fatigue life. The high values for the cube are most likely caused by the purely compressive loading it experiences, as opposed to the partial tension present in the other types of lattice structures [12,51]. The other unit cell types failed under similar conditions, with the lowest fatigue strength reported for the rhombic dodecahedron [12,20,51]. A clear three-stage strain accumulation pattern has been observed for all lattices [12,38,59], which corresponds well with another study on aluminum lattices [62]. The fatigue strength of the discussed unit cells was found to be in the range of 0.12 to 0.36 times the yield stress, which means that all unit cells showed a fatigue strength that was significantly lower than the fatigue strength of solid titanium specimens (i.e., 0.4 times the yield stress) [12,34,38,51,59]. This difference may be explained by the residual stresses, porosity, and the surface finish of the specimens [20]. At the moment of failure, all structures demonstrated a shear band of 45° perpendicular to the loading direction [20,34,35,38,40,51,54,55].

The above observations show that AM lattices are relatively sensitive to the relative density. Compared to the other unit cell types, changes in the relative density have the greatest effects on the diamond-based lattices. In contrast, the compressive properties of the cube are the least affected by the relative density [19]. Additionally, the AM process itself has been found to influence the mechanical properties of lattices. There are over a hundred process parameters that all have implications for the final product. For example, the laser exposure time and laser power have a positive relationship with the quasi-static mechanical properties of the resulting lattice structures [63]. Increasing both parameters also seems to diminish powder adhesion around the struts [64]. Furthermore, the slice thickness has been found to play an important role in the surface roughness of lattices [65]. Surface roughness causes stress concentrations, which may lead to micro-crack initiation [12,54]. The build direction has a huge effect on the mechanical properties as well, not only because grains tend to grow in the build direction, but also because horizontally oriented struts are generally of inferior quality as compared to vertically oriented struts [36,66]. Therefore, the AM process has been argued to introduce a certain level of anisotropy, especially in structures such as the cubic lattice [12,36]. This phenomenon has been supported by two studies in which a cubic lattice was tested at a 90-degree angle from the build direction. As a result, significantly lower compressive properties were found for those tested at a 90-degree angle from the build direction [48,49]. Diagonal struts are of intermediate quality, which explains the anisotropic behavior found in a study on diamond lattices [36]. In that sense, AM lattices are sensitive to the direction of the applied load with respect to the building direction [12,36,48,49].

Post-processing treatments have been proven effective in altering the mechanical properties of additively manufactured lattices [67]. Hot isostatic pressing (HIP, ASTM F2924 [68] class 2) has been found to decrease the internal porosity of the struts. This may not significantly affect the mechanical strength of the lattice structures; however, it does increase the ductility. That is particularly beneficial in dynamically loaded applications [36]. Furthermore, HIP has been shown to significantly improve the fatigue performance of lattice structures [69]. The ASTM F2924 [68] class 1 treatment, also referred to as the stress relieved condition, slightly increases the mechanical properties of the Ti-6Al-4V lattices; however, it also makes them more brittle [36,67].

### 3.1. Topology-Property Relationship in Non-Auxetic Mechanical Metamaterials

**Figure 8:**
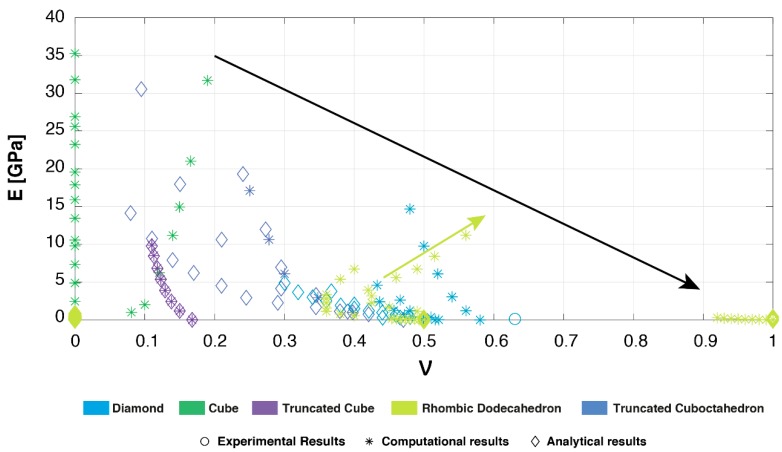
Young’s modulus versus Poisson’s ratio for all of the studied unit cells based on experimental, computational, and analytical data [19,21,22,33,35,42,46].

The unit cells in this review have been shown to exhibit a wide range of mechanical properties (Figure 6 and Figure 7), which can be explained on the basis of their topology and the resulting deformation mechanisms. The deformation mechanism of lattices is usually a combination of bending and buckling (compression) [70]. The type of mechanism dominating the overall deformation is mainly dependent on the strut orientation in the unit cell [40]. Struts that are aligned along the loading direction will deform by compression (stretching), while more inclined struts mainly deform through bending [40]. In literature, these are usually referred to as “stretch-dominated” and “bending-dominated”; however, we will use the term “compression-dominated” instead of “stretch-dominated” given that we are specifically focused on bone-mimicking metamaterials. Lattices dominated by the compression mechanism exhibited brittle characteristics and possessed a higher load bearing capacity [40,45,70]. Lattices dominated by the bending mechanism showed a more ductile behavior [45,70]. This explains why the cube, with most of the struts oriented in the loading direction, showed very high mechanical properties. The same holds true for the truncated cube, while the truncated cuboctahedron was located in the middle of the spectrum from compression- to bending-dominated. The diamond and rhombic dodecahedron belonged to the latter with a relatively low stiffness and high Poisson’s ratio (Figure 8). The rhombic dodecahedron was the only unit cell in this study with a transversely isotropic geometry, which mainly affected its Young’s modulus and Poisson’s ratio. All other unit cells showed similar mechanical properties, although the loading direction may be important due to AM-induced anisotropy.

The Poisson’s ratio describes the negative of the ratio of the transverse strain to the axial strain. In many cases, bending of the struts can increase the transverse strain of the discussed lattices. Therefore, given the previously mentioned deformation mechanisms of the unit cells, it is not surprising to find that the rhombic dodecahedron and the diamond unit cells exhibited the highest values of the Poisson’s ratio. The cube, in which none of the vertically oriented struts bend, had a Poisson’s ratio close to zero [19,42]. Most of the Young’s modulus-Poisson’s ratio duos in Figure 8 confirm this observation. Since an increase in the strut thickness (relative density) decreases the ability of the struts to rotate and bend around their vertices, the Poisson’s ratio and the relative density are negatively correlated. An exception to the above-mentioned observation is the rhombic dodecahedron, which according to Hedayati et al. (2017) shows a positive relationship between the Poisson’s ratio (ν_yx_) and Young’s modulus with increasing relative density (green arrow, Figure 8) [19]. Although a large range of Poisson’s ratios has been covered, a combination of high stiffness and high Poisson’s ratio has not yet been attained (black arrow, Figure 8). The truncated cuboctahedron seems to cover the biggest area of Young’s modulus-Poisson’s ratio duos, especially for higher values of stiffness. The rhombic dodecahedron can be considered the best choice for low stiffness applications requiring a certain change in volume.

For some of the unit cells, parametric studies have been performed to evaluate the effects of certain geometrical features on the mechanical properties. A computational study showed that the yield stress of the diamond, cube, and rhombic dodecahedron is greatly influenced by the cross-sectional shape of the struts [19]. A circular cross-sectional shape resulted in the highest mechanical property values, while a triangular cross-sectional shape showed the lowest [19]. Unlike the yield stress, the Young’s modulus has not been found to be affected by the cross-sectional shape of the struts [19]. The strut length did not influence the mechanical properties of these unit cells as long as the relative density remained constant [19]. Unfortunately, no parametric studies were found for the lattice structures based on the truncated cube and truncated cuboctahedron unit cells.

### 3.2. Design Recommendations for Biomedical Applications

Recently, metallic open-cell porous structures have been considered as an ideal substitute for the currently used solids in orthopedic implants [28]. These porous structures mimic the mechanical properties of bone, and therefore, could reduce the mechanical mismatch at the bone-implant interface. As a result, the physiological stress/strain distribution can be maintained, reducing the risk of implant loosening [29]. The unit cells in this review could potentially be applied in these bone-mimicking porous structures, given that their mechanical properties are in the range of those reported for bone [30,71,72,73]. The exact properties are highly dependent on the anatomical location and patient attributes such as age and bone quality [74]. Due to these variations, each and every one of the discussed unit cells may be successfully applied in different biomedical settings.

The cube and truncated cube showed a relatively high Young’s modulus in the range of those reported for cortical bone [30,71]. Therefore, they may be useful in high stiffness applications [19]. In contrast, the diamond exhibited mechanical properties similar to trabecular bone [30,71]. This unit cell might be applicable in implants that need to be more flexible and require a high Poisson’s ratio. The rhombic dodecahedron showed a relatively high yield stress, while its Young’s modulus was relatively low. Therefore, this unit cell might be used in applications that require low stiffness and high strength [19].

During daily activities, bone substitutes are subjected to cyclic loading particularly when they are used in load bearing locations. However, there is no consensus in the literature on the most optimal fatigue life for bone ingrowth. Yavari et al. (2013) argued that although sufficient mechanical support is necessary right after implantation, a high fatigue life may not be beneficial for bone ingrowth and long-term fixation [59]. The stiffness of the porous biomaterial will increase as a result of the additional mechanical support provided by the newly formed bone [75]. If the implanted porous biomaterial remains too stiff for an extended period of time, the regenerated bone will never get the chance to fully bear the load. Therefore, to design the most optimal bone substituting structure, we should better understand the fatigue loads that it will be subjected to. Ideally, the stiffness of the implant should gradually decrease with the same rate as the increase in the load bearing capacity of the newly formed bone. In this way, the load-bearing capacity of the bone-implant complex will be kept more or less constant.

Besides the optimal bone-mimicking mechanical properties and fatigue life, a lattice should exhibit certain biological characteristics. Porosity, pore size, and pore interconnectivity are important factors influencing the biological performance of metallic lattices [27,76,77]. Porous structures do not only promote vascularization, they will also facilitate the penetration of bone cells, which both contribute to bone growth [77]. Unfortunately, there is no consensus yet about the most optimal pore size. Some studies have claimed that the most favorable pore size for bone-formation ranges from 50 to 400 µm [76,78,79,80]. However, Taniguchi et al. (2016) found significantly more bone ingrowth in their 600 µm and 900 µm specimens as compared to the 300 µm structures after in vivo implantation in rabbit tibia [81]. Rumpler et al. (2008) argued that curvature is the driving force behind tissue regeneration, instead of the pore shape in general [82]. Furthermore, pore interconnectivity is important to supply nutrients and remove waste during cell growth [76]. Since the most optimal biological properties often lead to a reduction in the mechanical strength, it is extremely challenging to design a bone-substitute. Increasing the porosity may, for instance, promote bone ingrowth; but it will also decrease the load bearing capacity as more material is removed. In this case, the unit cell with the highest stiffness to weight ratio seems ideal; however, it may not be suitable in terms of pore shape.

### 3.3. Challenges and Limitations

In this review, we have tried to present a comprehensive library of the mechanical properties of five non-auxetic unit cells using experimental, computational, and analytical data available in literature. Additional techniques based on theories such as the effective medium theory (see e.g., [83,84,85]) are, thus far, only occasionally used. The use of such theories could help in better understanding the exact physical mechanisms behind the mechanical behaviors observed in experiments. In addition, the use of these theories could be helpful in establishing a relationship with other areas of relevant research. Overall, the mechanical properties of the diamond, cube, and rhombic dodecahedron unit cell have been extensively studied, meaning that there is more than one study on each mechanical property. The Poisson’s ratio is an exception, since it has only been studied using analytical and computational techniques. For all other quasi-static mechanical properties, a well-founded comparison could be performed. For both the yield stress and Young’s modulus, the amount of data for each of the unit cells is satisfactory for all methods. The first maximal stress was mainly determined using experiments with limited computational and analytical data. For the truncated cube and truncated cuboctahedron, the data was scarce, making their comparison less reliable. The data on the fatigue properties were also limited for all of the proposed unit cells, and the differences in data analysis made it hard to compare. Most studies reported the endurance limit as the ratio between the fatigue stress and the yield stress, while others used the fatigue stress to plateau stress ratio. Subsequently, some of the proposed unit cells failed before reaching 10^6^ loading cycles. Therefore, the fitted power law was extrapolated to properly compare the fatigue strength. This introduces a certain degree of inaccuracy, which could be overcome if the number of loading cycles for porous structures would be decreased. Another limitation is the fact that the effects of material type (independently and in combination with the effects of topological design) have not been considered, except for a few studies [13,23]. The titanium alloy Ti-6Al-4V has been widely applied in orthopedics because of its favorable properties such as corrosion resistance, high strength, and biocompatibility [86]. For some biomedical applications, other material types may be more beneficial; however, their examination falls beyond the scope of this review. Wauthle et al. (2015) argued that pure titanium might be more beneficial than titanium alloy for dynamically loaded porous implants, although Ti-6Al-4V still remains the strongest choice in static applications [87]. Recently, the isolated effects of material type on the quasi-static mechanical properties and fatigue behavior have been studied [13,23]. Results show that material type has a more dominant effect on the fatigue properties compared to the quasi-static mechanical properties [13,23].

Apart from the material type, the decision to focus on Computer-aided Designs (CAD) can also be considered a limitation. There are several other approaches to design the proposed micro-architectures. Each of the approaches can be classified in one of the following categories: image-based design [88,89], implicit surface modelling [90], and topology optimization [91]. Topology optimization in particular, might be an important tool for the development of porous meta-biomaterials. With this tool, the design process becomes a well-posed optimization problem [92]. Instead of looking for a unit cell type with favorable mechanical properties, it is possible to obtain the optimal micro-architecture by satisfying the mechanical and bone-mimicking requirements [90,93]. This sounds very promising; however, to be able to use this method, one should know what the desired properties are.

### 3.4. Potential Applications and Future Research

As mentioned earlier, the unit cells in this review could have great potential as meta-biomaterials. Their (unusual) mechanical, biological, and physical characteristics make them ideal substitutes for bone [4]. Functionally grading their composition may even enhance their beneficial properties, since the architecture of bone is hierarchical and consists of a dense outer layer (cortical bone) and a more porous interior (trabecular bone) [30,31,32]. An example of a functionally graded material could be an acetabular implant with a gradient relative density. In this case, the relative density of the unit cells is graded form very solid at the joint’s articulating surface to very porous at the bone-implant interface [94]. They have also been applied in the rationally designed meta-implants of Kolken et al. (2017), in which a combination of auxetic (negative Poisson’s ratio) and non-auxetic (positive Poisson’s ratio) meta-biomaterials were used [15]. This hybrid implant exerted pressure both on the lateral and medial surrounding bone, which cannot be achieved using conventional solid hip implants. Incorporating a meta-biomaterial with an even higher positive Poisson’s ratio, may result in an even better bone-implant interaction. The diamond or rhombic dodecahedron, for instance, which have demonstrated an extremely positive Poisson’s ratio, could be successfully applied on the medial side of the hybrid meta-implant. However, as described earlier, there is a lack of experimental data on the Poisson’s ratio. Therefore, it is not clear whether the Poisson’s ratio found in computational and analytical studies can be observed in actual experiments. Future studies should therefore focus on experiments to determine the Poisson’s ratio of these unit cell types. This could be done using digital image correlation (DIC) [95,96], which enables the examination of strains and displacements on the surface of a specimen [15]. The Poisson’s ratio could then be derived from these data.

Similar to the Poisson’s ratio, the fatigue properties have received insufficient attention. To actually implement these unit cells in meta-biomaterials for load-bearing applications, more research on their fatigue properties will be required. Most of the available studies focus on the compression-compression fatigue behavior, which has also been the focus of this review. Compression-compression is most often examined, because it is considered the most common mode of bone loading during daily activities [34]. However, for a comprehensive review on the fatigue life, it is important to also consider the bending and tensile fatigue behavior.

Although most of the geometries are defined by the angles between the struts, it would be really interesting to explore their behavior outside these aspect ratios. Adjusting the geometrical shape of the unit cells may have beneficial effects on the mechanical properties, especially for the Poisson’s ratio in the bending-dominated structures. Moreover, Zhao et al. (2016) have shown that increasing the internal angle of the rhombic dodecahedron enhances its fatigue life [51].

## 4. Conclusions

The five non-auxetic mechanical metamaterials considered in this review have been extensively studied; however, we can conclude that experimental data is lacking at least for some properties. This is especially true for the Poisson’s ratio, which has not yet been experimentally determined. In general, the cube was found to exhibit the highest mechanical properties; however, it also showed the lowest values of the Poisson’s ratio. Bending-dominated structures like the diamond and rhombic dodecahedron exhibit the lowest mechanical properties in terms of stiffness and strength; however, they possess the highest values of the Poisson’s ratio. To quantify the deformation behavior of these mechanical metamaterials, and assess their potential as bone-substituting materials, more experimental data will be needed on their Poisson’s ratio and fatigue life.
